# *Prescriptions for Connection*—Social Prescribing for Older People in Australia: Protocol for a Feasibility Study

**DOI:** 10.1111/hex.70506

**Published:** 2026-01-07

**Authors:** Heather Block, Candice Oster, Suzanne Dawson, Claire Gough, Gregory B. Crawford, Adelaide Boylan, Jan Angelo, Matthew Freeman, Rachel Milte, Helen Exley, Stacey George

**Affiliations:** ^1^ Caring Futures Institute, College of Nursing and Health Science Flinders University Adelaide South Australia Australia; ^2^ Adelaide Primary Health Network Adelaide South Australia Australia; ^3^ Adelaide Medical School University of Adelaide Adelaide South Australia Australia; ^4^ Port Adelaide Enfield Council Adelaide South Australia Australia

**Keywords:** aged, community participation, feasibility studies, loneliness, primary health care, quality of life, social isolation, social prescribing

## Abstract

**Background:**

Older people are living longer with unmet social needs that impact their health and wellbeing. Social prescribing programs connect individuals with nonclinical services to address these needs. While international programs have shown benefits for quality of life, health and healthcare utilisation, social prescribing research in Australia is limited.

**Objective:**

This study aims to design, implement and evaluate a social prescribing program to address the unmet social needs of older adults in Adelaide, South Australia.

**Research Design and Methods:**

This observational feasibility study will use a multiphase mixed methods process and outcomes evaluation. *Prescriptions for Connection*, a social prescribing program, will be co‐designed and implemented in six primary care practices and three council areas. Implementation outcomes will be evaluated using the Reach, Effectiveness, Adoption, Implementation and Maintenance Framework. Qualitative data collection, via focus groups for health and social care providers and interviews for older people, will be based on the Consolidated Framework for Implementation Research to explore barriers and facilitators, and explain outcomes.

**Conclusion:**

This study will provide novel evidence on the process, outcomes and feasibility of the *Prescriptions for Connection* program. Scalability, sustainability and modifications to the program will be explored for testing in a larger hybrid effectiveness‐implementation trial.

**Patient or Public Contribution:**

Older adults will participate in codesign workshops and provide feedback on the *Prescriptions for Connection* program. Community members will be recruited as volunteer community connectors to link older people to social activities. The national peak body for older Australians will be represented on the steering group.

**Trial Registration:** ACTRN12625000664448.

## 1 Introduction

Globally, the population is ageing rapidly, with estimations that by 2030, one in six people will be over the age of 60 years [1]. Population ageing is a major public health concern as increased longevity does not always correlate with good health [2, 3]. Older adults experience many underlying nonmedical causes of ill health, including social isolation and loneliness.

Loneliness (the painful feeling of being alone) and social isolation (a lack of social contact) are prevalent in older people [4], with approximately one in four experiencing either or both conditions globally [5, 6, 7]. Loneliness and social isolation are recognised as a global health priority [8], having similar effects on health to physical inactivity and obesity [9]. A recent study found persistent loneliness in adults aged 70 years and older was associated with an increased risk of cognitive decline and dementia, particularly for women [10]. Additionally, social isolation is shown to increase the risk of adverse physiological effects, including cardiovascular disease and ischaemic stroke [11]. Therefore, it is important to address loneliness and social isolation in older people to support healthy ageing [12]. There is no one‐size‐fits‐all approach to addressing loneliness and social isolation given the diversity of individuals, communities and their needs. Social interventions should be considered to be tailored to address the challenges faced by socially disadvantaged older adults, taking into consideration their preferences, cultural backgrounds and contextual factors, to reduce health inequities [13].

There is a growing movement towards social prescribing as a way to address loneliness, social isolation and other nonmedical needs [14, 15, 16]. Social prescribing is defined as a means for healthcare workers to connect individuals to a range of nonclinical services in the community to improve health and wellbeing [17]. There are many different ways in which social prescribing can be delivered [18]. This may be as simple as a health provider, such as a general practitioner (GP), also known as a family medicine practitioner, providing information on supports and services in the community. More intensive support is provided through holistic models that use “link workers” to assess needs, connect participants to services/community groups, and follow‐up over a period of weeks or months. Social prescriptions can come from multiple sources, most commonly through primary care. Participants can be prescribed a wide range of service supports, such as exercise or arts programs, social services such as homeless or food relief, and community groups. In link worker models, this role can be undertaken by people with a health background and those without such a background, with some programs involving students or volunteers [18].

There is evidence supporting social prescribing programs broadly in improving health and wellbeing, day‐to‐day functioning, social contacts and health‐related behaviours, and reducing healthcare demand [19, 20]. Within primary care settings, social prescribing has demonstrated improved health behaviours and self‐efficacy outcomes, such as reduced social isolation, improved quality of life, active living, social networks, self‐value, self‐confidence [21] and mental wellbeing [22]. A review of social prescribing in primary care by Griffiths et al. [23] found individuals engaging in a broad range of social prescribing activities demonstrated improvements in wellbeing, health status, quality of life, self‐management, physical activity and social connectedness. Despite these positive findings, the quality of evidence is lacking due to weak methodological designs in social prescribing primary studies [23].

Internationally, social prescribing programs have shown improvements in wellbeing, quality of life and reduced isolation and loneliness among older adults [24]. There is a paucity of evidence reporting the effectiveness of these programs addressing loneliness and social isolation in older people in Australia. One Australian program was identified that targeted loneliness and social isolation, however, it targeted adults in general and not older people specifically [15, 25]. Future social prescribing implementation should emphasise health equity and place‐based community development to address individuals’ outcomes and advance translation across health and societal systems [26, 27]. Thus, the preferred design, feasibility or effectiveness of implementing a social prescribing program for older people in the Australian setting is not known.

Social prescribing “requires collective action and collaboration among multiple sectors and stakeholders” [28]. It is considered a complex intervention that aims to connect and integrate services across fragmented healthcare, social and community sectors and, as such, it is important that the intervention be co‐designed with all key stakeholders to ensure adequate fit with existing systems, processes and needs [29]. Place‐based social prescribing interventions and models should consider codesign development to emphasise tailored social interventions with individuals within their communities. This project will involve collaboration between local government councils, Adelaide Primary Health Network (PHN), universities and general practices. Together, these organisations will work with communities, older people, health and social care providers to codesign, implement and evaluate a social prescribing program for older people experiencing loneliness and social isolation.

In the development and evaluation of complex interventions, such as social prescribing programs, guidance frameworks advise evaluations of not only efficacy and effectiveness, but also feasibility, and implementation, with consideration of the settings and context in which the intervention occurs [30]. This project will codesign, implement and evaluate the feasibility of a social prescribing program, called *Prescriptions for Connection*, to address the unmet social needs of older adults in primary care and local council settings.

### 1.1 Research Aim

To evaluate the feasibility of the *Prescriptions for Connection* program in improving quality of life, wellbeing and loneliness of older people in primary care and council settings.

### 1.2 Research Questions

1. What are the key components of the social prescribing program, *Prescriptions for Connection*, developed in collaboration with older people, health and social care providers?
2. What is the reach, effectiveness, adoption, implementation and maintenance (RE‐AIM) of *Prescriptions for Connection*?
3. What are the barriers and facilitators of *Prescriptions for Connection* in primary care and council settings?
4. What are the recommendations for a future trial in primary care and council settings?

## 2 Research Design and Methods

### 2.1 Design

This feasibility observational study will utilise multiphase mixed methods. A feasibility design is necessary to establish whether the novel social prescribing intervention could be successful if implemented [31].

The multiphase study will involve co‐designing, implementing and evaluating *Prescriptions for Connection*, a social prescribing program for older people in Adelaide, Australia. Reporting of findings will be consistent with the checklists of TIDieR [32], for the co‐designed social prescription intervention (*Prescriptions for Connection*), and CONSORT [33] for the overall study. The findings of this feasibility study will be used to inform future recommendations for implementation of social prescribing programs in primary care and council settings for older people in Australia [34]. This study has received ethics approval through the Flinders University Human Research Ethics Committee (Project number: 6789).

### 2.2 Theoretical Frameworks

Implementation evaluation of *Prescriptions for Connection* will be guided by the RE‐AIM [35, 36] and consolidated framework for implementation research (CFIR) [37] frameworks. The RE‐AIM framework is commonly used to evaluate implementation outcomes of new innovations, while understanding contextual factors that influence implementation [36, 38, 39]. RE‐AIM describes outcomes according to reach, effectiveness, adoption, implementation and maintenance [35, 36]. CFIR is a determinant framework that explores factors that influence implementation outcomes [37, 40, 41]. CFIR considers 48 constructs within five domains to influence implementation process and outcomes: intervention characteristics, outer setting, inner setting, characteristics of individuals and process [40, 41, 42]. Multilevel domains (individual adopters, organisational and policy factors) acknowledge the multiple interacting influences [40, 41, 42]. Use of the RE‐AIM and CFIR frameworks are frequently used to guide implementation process and evaluation [40, 41, 42]. As described by King et al. [43], this combination enables both:

1. A consideration of planning and evaluating the practice change of the implementation of the *Prescriptions for Connection* program to maximise its external validity, or ability of the evaluation findings to be generalised to other populations and settings using the RE‐AIM; and
2. An understanding of why implementation succeeded or not, identifying factors which can be modified to facilitate greater uptake and sustainability, using the CFIR.

### 2.3 Setting

This study will be undertaken across three local government areas located in northern Adelaide, South Australia: Port Adelaide Enfield, Walkerville and Prospect Councils. These three areas from inner to outer northern Adelaide have over 27,000 residents aged 65 years and over, with over 21,000 of these people living in Port Adelaide Enfield (Public Health Information Development Unit [PHIDU]) [44]. Those living in Port Adelaide Enfield show a high level of socioeconomic disadvantage, with 38% of people aged 65 and over living in the most socio‐economically disadvantaged quintile in Australia as measured by the Index of relative socioeconomic disadvantage [45]. Although Walkerville is significantly less disadvantaged socio‐economically, it has the second highest proportion of people aged 65 and over in the Adelaide Primary Health Network (Adelaide PHN) region (PHIDU) [44].

The Adelaide PHN will coordinate the involvement of primary care providers and provide expertise in the co‐ordination and integration of the *Prescriptions of Connection* program with local health care services in collaboration with the investigative team to improve quality of care, people's experience, and efficient use of resources.

**Figure 1:**
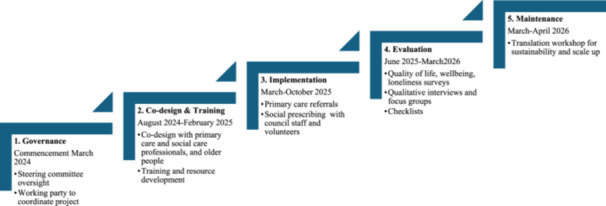
Prescription for Connection project phases and timeframe.

A postdoctoral researcher will be responsible for coordinating the project and conducting the evaluation including data collection and analyses. Two social prescribing officers will be employed staff through the Port Adelaide Enfield Council with experience in health and wellbeing community service programs. Social prescribing officers will oversee navigation of the social prescribing program, including receiving and triaging referrals, contacting, consenting and goal setting with older people participating in the program across the three council areas. In addition, they will provide supervision, training and support to Community Connector volunteers and will assist in data collection. Community Connectors will be registered volunteers from the participating councils, and will support older adults' social engagement in groups and activities during the program. The project will be completed in five phases as detailed in Figure 1.

### 2.4 Phase One—Project Governance

A steering group of key stakeholders will be formed to provide oversight and expertise throughout the study. Steering group stakeholders will include project partners from Adelaide PHN, Council on the Ageing South Australia (COTA SA), participating council leaders, funding body representatives and chief investigators. The steering group will be responsible for the governance of the project. A project working group will also be formed to include project staff, the investigator team, and a GP. The working group will oversee coordination and conduct of the project phases.

### 2.5 Phase Two—Codesign and Development

#### 2.5.1 Codesign

The *Prescriptions for Connection* social prescribing program will be informed by recent literature emphasising the need for contextually tailored social prescribing to address nonmedical needs for older adults [18]. The program will be co‐designed to meet the needs of the community, with involvement from primary care and social care professionals and older people.

Two 2‐h codesign workshops will be held to determine the process and resources required for *Prescriptions for Connection*. One workshop will be held with 15–20 primary care and social care professionals, including GPs, practice nurses, practice manager, general practice facilitators, professionals and leaders from social programs within Councils. A second workshop will be held with 15 older people, aged 60 years and older residing in the participating council areas.

Codesign will be underpinned by Trischler's seven step codesign process; providing comprehensive guidance on steps and tools for engagement, ensuring the voices, experiences and needs of participants remain central during codesign [46]. The application of the seven codesign steps is described in Table 1.

**Table 1:** The seven‐step codesign process guided by Trischler et al. [46].

Step	Description
Resourcing	An understanding of the problem to be addressed will be gained through literature reviews on this topic. A steering committee of stakeholders (described in Phase One—Project governance) will guide the project governance.
Planning	Planning will be an iterative process with the steering committee and project working group to determine the goals and outcomes of codesign. Regular working group meetings were held to reflect on each workshop and plan for the next.
Recruiting	The steering committee and working group will conduct stakeholder mapping to identify relevant participants and strategies for recruitment to the codesign workshops.
Sensitising	Sensitising will prepare workshop participants about the concept of social prescribing, the design task and trigger reflections on the topic.
Facilitation	Codesign tools will be used to foster creativity in individual activities and group discussions, such as workbooks, preference rating scales, butchers paper, sticky notes, coloured pens. Small group discussions will be facilitated by a member of the working group. Audio recordings of discussions, written ideas and notes will be analysed to identify themes for components of the social prescribing program.
Reflecting	Reflection will be undertaken with steering committee and working group members to plan and explore feasibility and realisation of the proposed model of care.
Building for change	Open dialogue with steering committee stakeholders will prepare for feasibility of implementing the designed to confirm the *Prescriptions for Connection* program before implementation and evaluation phases.

#### 2.5.2 Training and Development

Following the codesign of the *Prescriptions for Connection* program, training and education will be developed for general practice and primary care professionals to identify and refer older people to the program. Training and staff development will be provided free of charge to Council staff and volunteers involved in navigating and connecting older people to social activities. This will include training in the social prescribing purpose, model, processes, goal setting, peer support and mental health first aid. Council staff will develop a directory of social services within each local council area to support navigation and involvement in social participation for older people involved in *Prescriptions for Connection*.

### 2.6 Phase Three—Implementation

The implementation of the *Prescriptions for Connection* program will be informed by Phase Two—Codesign outcomes. Through codesign, processes and materials will inform the implementation of *Prescriptions for Connection* as described in Table 2.

**Table 2:** Co‐designed referral pathways, processes and supporting materials for implementing prescriptions for connection.

Program component	Details and implementation
Referral sources	GP, community health services, general practice team members
Participant eligibility	Older people aged 60 years and above living in the community. Experiencing social isolation, loneliness, or seeking more social connection.
Identification methods	Professional judgement or validated screening tool
Referral mechanisms	Phone call, referral form, self‐referral
Volunteer engagement and support	Where is the best place to meet, transport access, goal setting, supporting and training volunteers, number of sessions with volunteer community connector.
Postprogram feedback process	Whether feedback is required to referrer, and if so, what detail of information is provided.

#### 2.6.1 Prescriptions for Connection Program Participants

Community dwelling older people aged 60 years and over will be eligible to participate in the social prescribing program. Further eligibility and exclusion criteria will be determined through codesign workshops. We will aim to recruit up to a maximum of 200 older people for the social prescribing program. Participation in *Prescriptions for Connection* also involves completing the research evaluation of quality of life, mental wellbeing and loneliness outcome measures. Primary care professionals and those involved in delivering the social prescribing program will be invited to participate in qualitative evaluation described below.

### 2.7 Phase Four—Evaluation

The evaluation will explore the feasibility, impact and uptake of the *Prescriptions for Connection* program, and barriers and enablers across the different primary care and council settings. The RE‐AIM [36] and CFIR [37] frameworks will be used to guide the evaluation of the implementation of *Prescriptions for Connection*. Table 3 provides a description of the RE‐AIM domains and the application in the evaluation. Table 4 describes the CFIR domains most relevant to the evaluation and the interview guide for older people and referrers, key program staff and volunteers, to identify the barriers and facilitators to effective implementation.

**Table 3:** Evaluation of prescriptions for connection guided by RE‐AIM framework.

Framework domain	Defined	Data measured	Measurement
**Setting‐level impact of prescriptions for connection**			
Reach	The proportion and characteristics of older people who participate in the social prescribing program.	– Number of referrals received – Number of older people who consent and participate – Demographics of older people participating in the program – Number of older people who decline or withdraw participation – Number of visits with Community Connector and record of connected social activities for each older person	– Checklist of program referrals and engagement – Checklists of community connection – Demographics form
Effectiveness	The degree to which the social prescription program produced the intended or unintended changes in outcomes.	The following outcomes measured at baseline, 3 months and 6 months: – Quality of life – Mental wellbeing – Loneliness scores	– QOL‐ACC and EQ‐5D‐5L – WEMWBS – UCLA (3 item)
**Setting‐level factors that support successful implementation**			
Adoption	The number and proportion of social prescribing services that older people engaged with during the program.	Number of people referred, engaged and completed the program	– Checklist of program referrals and engagement – Checklist of– community connection
Implementation	The factors for successful implementation of the social prescribing program, with any adaptions or modifications.	– Interviews with older people who participated in the program – Focus groups with general practice referrers, council staff and volunteers.	– Focus groups and interviews
Maintenance	The degree to which the social prescription program is adopted, maintained and opportunities for scale‐up	Translation workshop to evaluate the scalability of the program	– Intervention Scalability Assessment Tool [47]

**Table 4:** Qualitative data to be explored guided by CFIR domains.

CFIR domain	Defined	Qualitative data to be explored
Implementation process	Stakeholder's engagement and decision‐making throughout implementation	Described process of referrals and process of meeting with participants, and connection to community social activities. Activities and strategies used to support implementation.
		Factors that support or hinder implementation in usual work processes.
Intervention characteristics	Intervention characteristics relating to the innovation that influence successful uptake	Advantages and disadvantages of *Prescriptions for Connection*; tailoring and adaptations required to fit within the council and community setting.
		Likelihood and factors required to continue implementation and participation in Prescription for Connection.
Outer setting	External factors influencing successful implementation	To what extent *Prescriptions for Connection* meet the needs of the community? Values, policy, quality benchmarking that support or hinder implementation.
Inner setting	Aspects of the organisation affecting successful implementation	Organisational resources, supports, relational connections, infrastructure, systems required to adopt *Prescriptions for Connection*. Were these factors effective in supporting adoption?
Characteristics of individuals	Characteristics of individuals that influence successful implementation	Motivation and confidence to implement and participate in *Prescriptions for Connection*

### 2.8 Participants

#### 2.8.1 Codesign Participants

Codesign workshop participants will include primary care and social care professionals (GPs, practice nurses, practice managers, general practice facilitators, professionals and leaders from social programs within Councils), and older people, aged 60 years and older residing in the participating council areas. Primary care and council professionals will be recruited for workshops through our existing networks of stakeholders with Adelaide PHN, Council partners and general practices within the participating council regions. Primary care and council professionals will be remunerated with $150AUD gift card for participation in the workshop. Port Adelaide Enfield, Walkerville and Prospect councils will assist in recruiting older people from their communities to participate in a workshop. Older people will be remunerated with $50AUD gift card for participation in the workshop. All participants involved in codesign workshops will provide written informed consent.

#### 2.8.2 Prescriptions for Connection Participants

Older people who participate in the social prescribing program will be aged 60 years and above, living in the community (not residential care), and willing and able to participate in social activities. Older people will be recruited into the program by referring health professionals. The social prescribing officers will triage referrals and gain permission to be contacted by the researchers for outcome measures and qualitative data collection. All participants will provide written informed consent.

### 2.9 Data Collection

A combination of quantitative and qualitative data collection approaches will be used.

#### 2.9.1 Quantitative Data

The primary focus is on evaluating the feasibility of implementation of the *Prescriptions for Connection* program, which will be assessed through checklists, recording program referrals and participant engagement, dropouts and withdrawals and checklists of community connection capturing social activities for older people. Social prescribing officers will record numbers of referrals received, referring organisations, reasons for referrals, numbers of older people who consent and participate in the program, and numbers of older people who are ineligible, decline or withdraw participation. Community Connectors will use a checklist to record number of contacts with each older person and types of social activities the older person was connected with.

Secondary outcomes will compare the impact of the social prescribing program on the quality of life, mental wellbeing and loneliness of older people at their commencement of the social prescribing program (baseline), compared to 3 months and 6 months later. Outcome measures will be collected by a member of the research team via Qualtrics surveys either in person or via telephone with the *Prescriptions for Connection* participants., Outcome measures used will be:

1. Quality of life will be measured with two instruments, the quality‐of‐life‐aged care consumers (QOL‐ACC) and the EuroQol five‐dimension health‐related quality of life instrument (EQ‐5D‐5L). The QOL‐ACC is a self‐administered quality of life questionnaire covering six dimensions (mobility, pain management, emotional well‐being, independence, social connection and activities) developed from first principles with older people through extensive qualitative research. The QOL‐ACC has been extensively validated for use in older people [48] including self‐report ability for older people with cognitive impairment [49]. The EQ‐5D‐5L is a widely used quality of life measure which is also able to be applied in economic evaluation. The descriptive system covers five dimensions including mobility, self‐care, usual activities, pain and discomfort and anxiety and depression [50]. The EQ‐5D‐5L also contains a visual analogue scale (VAS), where participants are asked to rate their general health today on a scale between 0 (indicating the worst health you can imagine) and 100 (indicating the best health you can imagine). The EQ‐5D‐5L has been applied extensively in many countries, and population ‘norms’ are available for comparison [51]. The EQ‐5D‐5L is the most widely used preference‐based measure across the health system [51], however the QOL‐ACC performs well in aged care settings [52]. Both the QOL‐ACC and EQ‐5D‐5L will be used for this study to determine how to two measures perform for older people in community settings.
2. Mental wellbeing will be measured using the Warwick‐Edinburgh Mental Well‐Being Scale (WEMWBS). The WEMWBS is a validated instrument for measuring mental wellbeing for older adults [53, 54], and previously used in social prescribing research [55]. The WEMWBS consists of 14 positively worded statements of mental health answered using a five‐point Likert scale. The Likert scale represents a score for each item, giving a minimum score of 14 (lower level of mental wellbeing) and maximum score of 70 (higher level of mental wellbeing) [54].
3. The UCLA Three‐Item Loneliness Scale is a validated instrument used among community‐dwelling adults, measuring general feelings of loneliness using three items with simplified response categories [56, 57].

Maintenance of the *Prescriptions for Connection* Program will be measured using the Intervention Scalability Assessment Tool (ISAT) [47]. The ISAT contains five domains covering aspects of scale‐up context and proposed implementation requirements, with a summative assessment scoring a readiness for scale‐up assessment [58].

#### 2.9.2 Qualitative Data

Implementation determinants will be explored through interviews and focus groups conducted by a member of the research team. Primary care professionals, council staff and volunteers involved in referring and delivering *Prescriptions for Connection* will be invited to participate in qualitative focus groups at a negotiated time convenient to them. All participants in qualitative data collection will provide written informed consent. Three focus groups will be conducted, one with primary care professionals including GPs, practice and community nurses, practice managers and care coordinators, one with local Council staff, and another with Community Connector volunteers, aiming for a total of *n* = 20 participants. Focus groups will explore perspectives of referring, delivering, implementing and sustaining *Prescriptions for Connection*. Interviews will be conducted with a subset of *n* = 20 older people to gain their experiences of the social prescribing program. Interviews will be audio recorded and professionally transcribed for analysis.

#### 2.9.3 Data Analysis

Quantitative data will be analysed using SPSS Version 28 statistical software [59]. Checklist numerical data and participant demographics will be analysed descriptively reporting number, frequencies, percentages, mean and SD, median and IQR. Quality of life, mental wellbeing and loneliness scores will be analysed descriptively as utility scores, mean and SD. Utility scores for the QOL‐ACC and EQ‐5D‐5L will be weighted using the Australian value set. To evaluate the difference between time points for quality of life, mental wellbeing and loneliness, pre‐ and postvariables will be analysed using paired *t*‐tests or nonparametric equivalent. Where comparisons are made across more than two time points, corrections to *p*‐values will be made using the Holm‐Bonferroni method. Chi‐squared test for independence will be used to test for any relationship between age group and responses to the outcome measures. *p*‐values of < 0.05 will be considered statistically significant.

Qualitative data from interviews and focus groups will be analysed in Nvivo [60] using thematic codebook analysis according to Braun and Clarke's criteria [61] with findings mapped to the CFIR framework [37] to identify barriers and enablers to implementation.

### 2.10 Phase Five—Maintenance

A translation workshop will be undertaken with primary care and council stakeholders (*n* = 15). The implementation and evaluation findings of the social prescribing program will be presented to workshop participants, who will be asked to individually evaluate scalability of the social prescribing program using the ISAT [47, 58]. Ratings will be presented to the group, and a discussion will be led to explore sustainability and modifications to the program, and strategies for scale‐up, translation, and testing in larger implementation trials. Workshop outcomes will inform a scalable, sustainable model of social prescribing for older people and associated social prescribing program logic for broader implementation.

## 3 Discussion

Social prescribing programs have been found to address loneliness and social isolation, improve health and wellbeing, day‐to‐day functioning, social contacts, health‐related behaviours and healthcare demands [14, 15, 16, 19, 20]. However social prescribing models are in their infancy in Australia. This study is innovative and important to address loneliness and social isolation in older Australians to support healthy ageing [12, 18]. Through tailored design, implementation and evaluation, *Prescriptions for Connection*s is an innovative approach, which has the potential for substantial impact to address the quality of life, wellbeing and loneliness for older people in Adelaide.

Healthy ageing is a priority of government sectors at all levels (Commonwealth, State, Local) in Australia, however, lack of integration between these sectors hinders quality of care to older people [62]. Codesign and collaboration across primary care services, local government Councils, Adelaide PHN and universities ensures robust development of the *Prescriptions for Connection* social prescribing program, that is tailored to suit individuals within the context of their communities. Through codesign, this project builds capacity to implement contextually relevant social prescribing, with opportunities for sustainability and broader scale‐up of the program and evaluation. New evidence gained from this study will explore feasibility and address the impact on nonmedical factors on older people's quality of life, wellbeing and loneliness. Broader adoption of social prescribing has the potential for significant improvements in older Australian's health and quality of life as they age, and to reduce the demand on health systems.

There is value in conducting a feasibility study in the implementation of a social prescribing approach in the Australian setting, given the complexity of multiple sector involvement across primary and local governments. In this study, the use of implementation frameworks (RE‐AIM and CFIR) will provide an in‐depth understanding of factors affecting successful implementation to support a future effectiveness trial. Councils and primary care professionals are the level of government closest to the community and have a key role in promoting thriving communities. A strength of this project is that the concept for trialling social prescription in this target setting originated from the local council. Other innovative examples of tackling health issues in community settings that have been driven by non‐health organisations include social workers in libraries [63].

Additionally, this study design is consistent with recent literature by Dingle, Aggar [26] calling for social prescribing implementation research in Australia and the United Kingdom that uses: (a) multilevel or systems theory frameworks to inform programme design and implementation (RE‐AIM and CFIR); (b) methods developed in collaboration with participants and service providers, involving: COTA representation, primary care providers, councils, researchers, and primary health networks; (c) a core set of outcome measures developed and complemented by framework‐specific measures through literature search, program theory, RE‐AIM and CFIR [4]; factors at multiple levels could be included to ensure a comprehensive understanding of the experience and value of social prescribing (RE‐AIM and CFIR); (d) semistructured interviews will focus on understanding barriers and enablers of engagement in social prescribing in marginalised populations; and (e) focus groups will explore link workers' and community workers' experiences of social prescribing. Thus, the findings from this study evaluating the feasibility of the *Prescriptions for Connection* program will contribute to the international knowledge base for social prescribing.

### 3.1 Strengths and Limitations

This study has been robustly designed and underpinned by theoretical implementation frameworks, with collaboration between local government councils, the primary care sector and clinical researchers with expertise in social prescribing and implementation of complex interventions. Study findings will describe a novel integration approach of social prescribing across primary care and social care systems. A potential limitation of this study is volunteer burden on community connectors which could influence sustainment and feasibility. This is being mitigated by Council led volunteer training processes and regular volunteer support sessions.

## 4 Conclusion

This study will design, implement and evaluate the first social prescribing program integrated with primary care GPs, councils and volunteers in Adelaide, Australia. If the program demonstrates feasibility and significant improvements to quality of life, wellbeing, or loneliness among older adults, it will provide an innovative approach to treating socially isolated older people with frequent GP consultations for nonmedical needs. Understanding of the implementation outcomes and opportunities for broader adoption will inform how this program can be best translated into routine primary care for older people across community settings. Further opportunity for scale‐up and trials will be needed.

## Author Contributions

**Heather Block:** investigation, project administration, data curation, formal analysis, visualisation, writing – original draft preparation. **Candice Oster:** conceptualisation, formal analysis, funding acquisition, investigation, methodology, writing ‐ review and editing. **Suzanne Dawson:** conceptualisation, formal analysis, funding acquisition, investigation and methodology, writing – review and editing. **Claire Gough:** project administration, writing – review and editing. **Gregory B. Crawford:** conceptualisation, funding acquisition, investigation, writing – review and editing. **Adelaide Boylan:** conceptualisation, funding acquisition, investigation, writing – review and editing. **Jan Angelo:** project administration, resources, writing – review and editing. **Matthew Freeman:** formal analysis, writing – review and editing. **Rachel Milte:** formal analysis, methodology, writing – review and editing. **Helen Exley:** project administration, resources, writing – review and editing. **Stacey George:** conceptualisation, funding acquisition, investigation, methodology, project administration, supervision, validation, writing – review and editing.

## Ethics Statement

This study has received ethics approval through the Flinders University Human Research Ethics Committee (Project number: 6789).

## Conflicts of Interest

The authors declare no conflicts of interest.

## Data Availability

Research data are not shared.
